# Involvement of an FTO gene polymorphism in the temporomandibular joint osteoarthritis

**DOI:** 10.1007/s00784-021-04278-9

**Published:** 2021-11-23

**Authors:** Ryota Takaoka, Kotaro Kuyama, Hirofumi Yatani, Shoichi Ishigaki, Hiroki Kayashima, Yukiko Koishi, Takafumi Kato, Hiroshi Egusa, Yuka Uchiyama, Atsutoshi Nakatani, Hiroaki Shimamoto

**Affiliations:** 1 Department of Fixed Prosthodontics, Osaka University Graduate School of Dentistry, 1-8, Yamadaoka, Suita, Osaka 565-0871 Japan; 2 Department of Oral Physiology, Osaka University Graduate School of Dentistry, 1-8, Yamadaoka, Suita, Osaka 565-0871 Japan; 3 Division of Molecular and Regenerative Prosthodontics, Tohoku University Graduate School of Dentistry, 4-1, Aobakuseiryoumachi, Sendai, Miyagi 080-0872 Japan; 4 Department of Oral and Maxillofacial Radiology, Osaka University Graduate School of Dentistry, 1-8, Yamadaoka, Suita, Osaka 565-0871 Japan

**Keywords:** FTO, Osteoarthritis, Temporomandibular joint, Obesity, Risk factor

## Abstract

**Objectives:**

The FTO gene has been reported as an obesity-associated gene and is also considered a risk gene for osteoarthritis (OA). However, its exact function is unclear, and there is conflicting evidence on the involvement of FTO polymorphisms in OA via obesity. The purpose of this study was to determine the effects of FTO polymorphism rs8044769 alleles on OA in the temporomandibular joint (TMJ), which is minimally affected by body weight.

**Materials and methods:**

A total of 324 TMJs (113 with OA and 211 without OA, serving as controls) from 162 Japanese patients with temporomandibular disorders and undergoing MRI examination were analyzed. Genotyping was conducted, and multivariate analysis was performed after adjusting for the effects of age, sex, body mass index, and TMJ disc abnormalities.

**Results:**

Mean age, BMI, and sex did not differ between the TMJs with OA and the TMJs without OA, but a significant difference was found for positional and dynamic disc abnormalities (*P* < 0.05). The allele frequency of FTO polymorphisms also differed significantly between the TMJs with OA and the TMJs without OA (*P* = 0.011). Moreover, logistic regression analysis showed no significant association between BMI (*P* = 0.581) and the occurrence of TMJOA but also indicated that the CC allele of rs8044769 is a risk factor for TMJOA (*P* = 0.040).

**Conclusions:**

Our results show that rs8044769 in the FTO gene might be involved in TMJOA.

**Clinical relevance:**

The present study provides a basis for a deeper understanding of the mechanism underlying degenerative skeletal diseases and the more effective selection and development of treatment strategies based on the patients’ genetic characteristics.

**Supplementary Information:**

The online version contains supplementary material available at 10.1007/s00784-021-04278-9.

## Introduction

The FTO gene, which encodes 2-oxoglutarate-dependent nucleic acid demethylase [1], has been reported as an obesity-associated gene [2, 3] and is expressed in many tissues, including the cerebral cortex, hypothalamus, pituitary, and muscle [4]. The FTO gene was the first gene to be cloned after the identification of a fused toe (Ft) mutation resulting in a 1.6 Mb deletion in mouse chromosome 8 [5, 6]; however, its function remains unknown. FTO mRNA in the arcuate nucleus of the hypothalamus is regulated by dietary restrictions, suggesting its involvement in energy homeostasis [7]. In recent years, the FTO gene has been attracting attention in the field of orthopedics as a risk gene for osteoarthritis (OA) [8–11], and a genome-wide association study by Zeggini et al. reported that the C allele of an FTO SNP (rs8044769) was strongly associated with OA in females [12]. Their excellent discoveries that have powerful influence on the subsequent studies have undoubtedly provided a great understanding of OA. However, whereas many studies conclude that the rs8044769 polymorphism affects OA through obesity [12, 13], some believe that this hypothesis is not sufficiently proven because of the difficulty in confirming the causal relationship between obesity and OA [14]. The mandibular condyle, which develops due to the same intracartilaginous ossification as the knee joint, has a structure that hangs from the skull. There are few studies on the relationship between body mass index (BMI) and TMJOA, and the absence of this relationship was reported by Winburn et al. [15]. Furthermore, due to the weak relationship between occlusal force and BMI [16], TMJ is minimally affected by body weight.

Degenerative lesions mainly indicate OA in the hard tissue of the joint, which develops in all joints of the human body, including the TMJ in the orofacial region. TMJOA is a sub-pathological condition of TMD and is considered the end stage of the disease [17, 18]. Its clinical symptoms include at least one joint noise (mainly crepitus), jaw dyskinesia, and pain in the TMJ. In addition, it has been reported that TMJOA clinical symptoms significantly reduce the quality of life [19], and many refractory patients do not obtain the desired therapeutic effects even if they receive appropriate treatment. Nonetheless, the pathophysiology of TMJOA is highly complex, and its definitive etiology has not yet been established.

Investigation of FTO in the mandibular condyle allows for an understanding of the effects of FTO SNPs on OA while adjusting the effect of body weight on joints to the fullest extent. We hypothesized that the FTO gene (rs8044769) is involved in the development of OA in the mandibular condyle, regardless of the body mass index (BMI). Therefore, in this study, we aimed to elucidate the effects of rs8044769 allele on mandibular condyle OA using multivariate analysis after adjusting for the effects of age, sex, BMI, and temporomandibular joint (TMJ) disc abnormalities.

## Methods

### Subjects

**Fig. 1:**
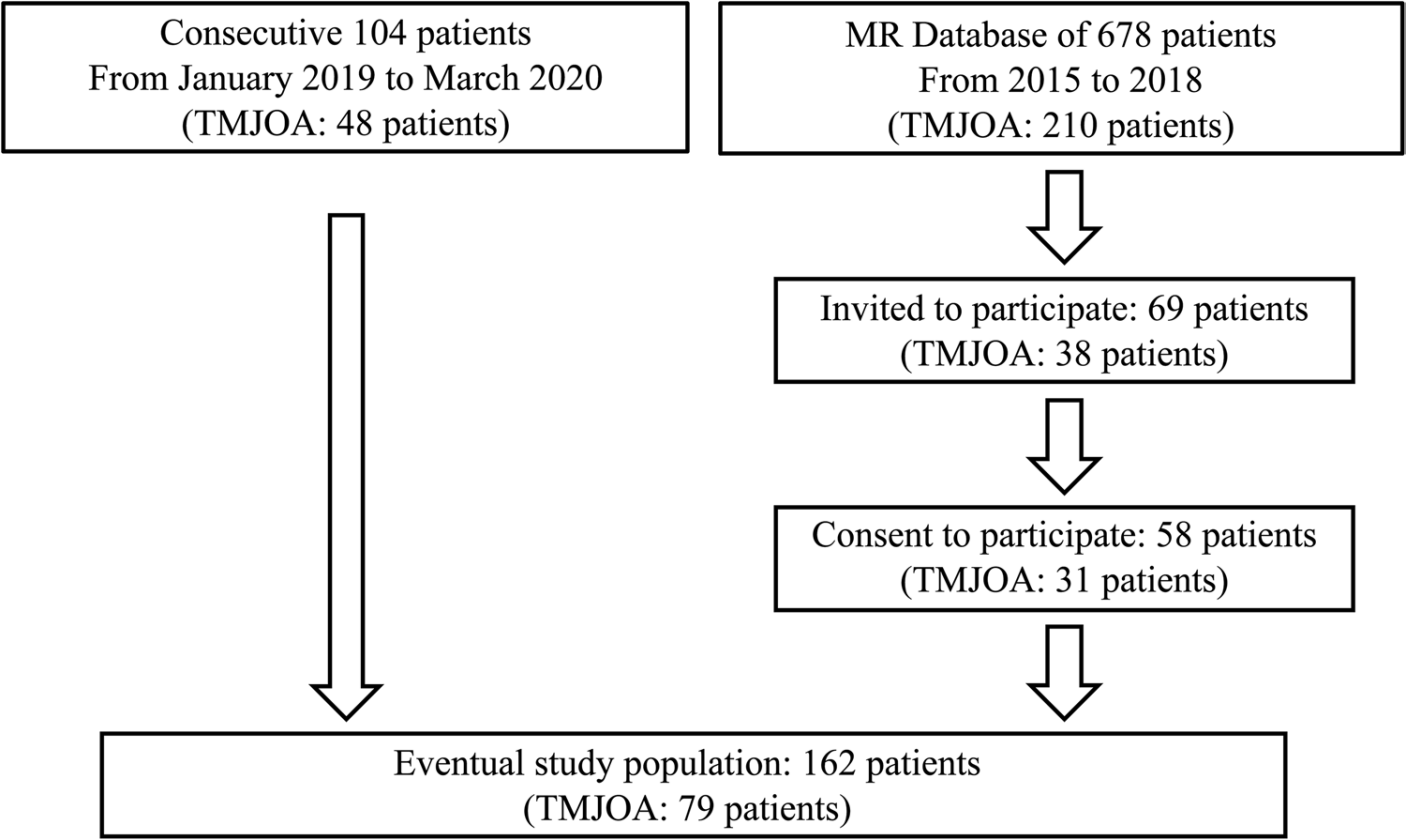
Recruitment scheme. TMJOA, temporomandibular joint osteoarthritis

One hundred and sixty-two Japanese patients (average age 57.0 ± 12.2 years; 32 males, 130 females) who visited the Osaka University Dental Hospital with symptoms of temporomandibular disorders (TMDs) and underwent MRI examination were analyzed in this study (Fig. 1). Patients under 30 years of age, those without MRI scans, and those presenting metal and motion artifacts on MRI scans were excluded from the study. Of the 162 patients, 104 were consecutive patients who visited the hospital between January 2019 and March 2020 (48 patients with TMJOA, 56 patients without TMJOA). Samples from the remaining 58 patients were extracted from the MR database of 678 patients who visited the hospital between 2015 and 2018 and underwent TMJ MRI examination. Of these 58 patients, 31 had severe degenerative bone changes in the mandibular condyle, and 27 had normal TMJ with no anatomical abnormalities, such as disc displacement and degenerative bone change. Written informed consent to participate in the study was obtained from all subjects. All procedures performed in studies involving human participants were in accordance with the 1964 Helsinki declaration and its later amendments or comparable ethical standards and conformed with the ethical standards of the institutional and/or national research committees. The present research complied with the STROBE statement. The study procedures have been approved by the Ethical Review Board of Osaka University (H30-E11).

MRI was obtained by utilizing a 1.5 T MR imaging scanner (SignaHDxt 1.5 T; GE Healthcare) equipped with a TMJ surface coil. All participants underwent imaging in axial, sagittal, and coronal planes utilizing fast spin-echo sequences. Axial T1-weighted images were obtained as the localizer as follows: TR = 150, TE = 4.2.The sagittal planes were oriented perpendicular to the long axis of the condylar head. Sagittal and coronal proton density-weighted images at the mouth closing were oriented as follows: TR = 2500, TE = 20, ETL = 8, and NEX = 2. Sagittal and coronal fat-suppressed T2-weighted images at the mouth closing were acquired as follows: TR = 2000, TE = 85, ETL = 16, and NEX = 3. Proton density-weighted images at the mouth closing and opening were acquired to evaluate disc displacement and the presence of reduction as follows: TR = 800, TE = 24, ETL = 4, and NEX = 2. The other parameters were as follows: 22.2 × 10 cm field of view, 256 × 160 matrix size, 3-mm section thickness.

Height and weight self-reported by the patients at the time of MRI were recorded. BMI was calculated by dividing the patient’s body weight by the square of their height.

### Diagnosis of OA and internal derangement of the TMJ

**Fig. 2:**
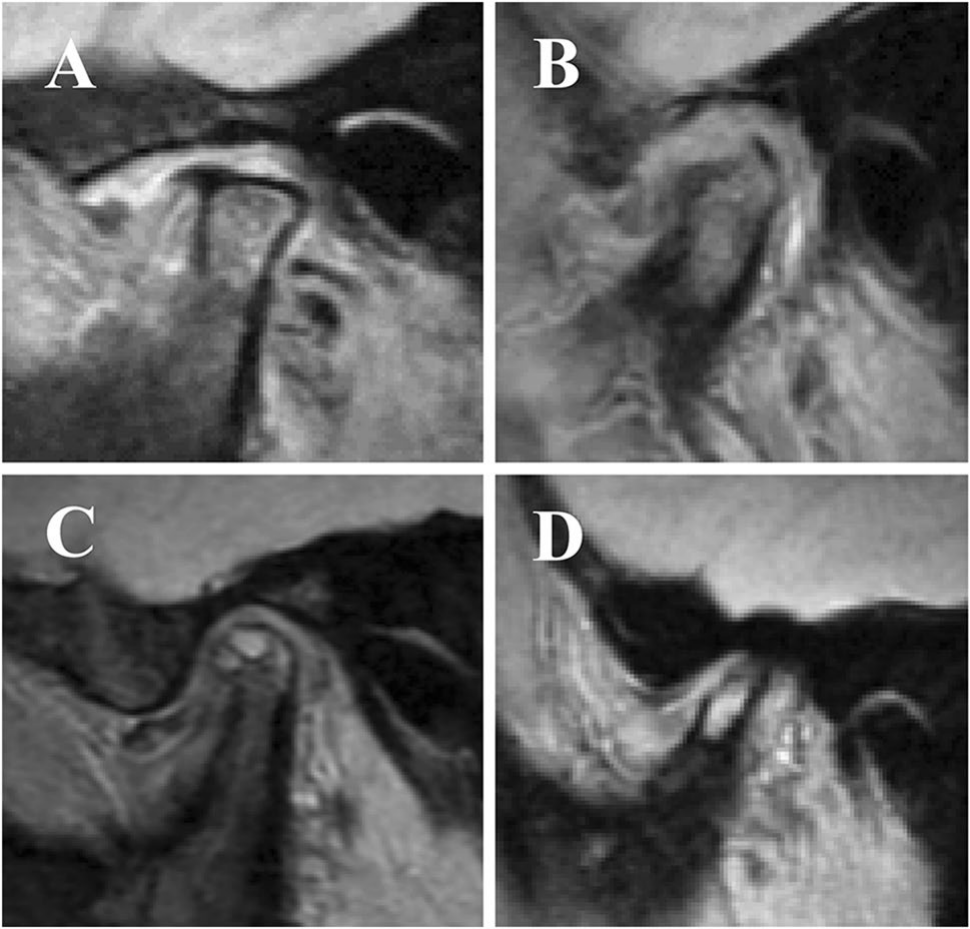
Examples of osteoarthritis on MRI. **A** Osteophyte. **B** Erosion. **C** Subchondral cyst. **D** Atrophic deformity

Two oral and maxillofacial radiologists performed diagnosis of OA and internal derangement of the TMJ upon MRI examination. The presence or absence of the following was recorded as degenerative bone changes in the mandibular condyle from the proton density-weighted images of the sagittal and coronal planes and T2-weighted images: osteophyte (marginal hypertrophy with sclerosing borders and growth of bone tissue from the bone surface, Fig. 2A), erosion (loss of continuity of cortical bone, Fig. 2B), subchondral cyst (high signal areas for cysts under the joint surface in T2-weighted images, which are different from normal bone marrow, Fig. 2C), and atrophic deformity (its sagittal plane has a thin rod-like appearance, and its coronal plane has a mandibular condyle that is less than half the size of the normal mandibular condyle inferred from the mandibular fossa, Fig. 2D). The diagnostic criteria for OA were at least one imaging finding of osteophytes, erosion, atrophic deformity, and subchondral cysts. Generalized sclerosis was excluded from OA because of the difficulty of accurate diagnosis by MRI [20]. All 324 mandibular condyles were classified into the TMJs with OA and the TMJs without OA. The positional abnormalities of the articular disc were classified as normal, sideways displacement, partial anterior displacement, partial anterior and sideways displacement, complete anterior displacement, or posterior displacement [21–23].

1. Normal: In a closed-mouth position, the top of the condyle was located underneath the intermediate thinnest portion of the disc, and the disc had a biconcave shape in all sections of sagittal imaging slices.
2. Sideways displacement: In the coronal imaging plane, the disc was laterally displaced over the medial or lateral poles.
3. Partial anterior displacement: Disc was anteriorly displaced in the lateral or medial part of the disc, whereas the top of the condyle was positioned underneath the intermediate zone of the disc in the contralateral portion.
4. Partial anterior and sideways displacement: Combination of partial anterior displacement and sideways displacement.
5. Complete anterior displacement: The posterior band was located anterior to the anterior surface of the condylar head in all sagittal slices.
6. Posterior displacement: In sagittal slices, the disc is posterior to the condyle, and enlargement of the posterior band appears.

**Fig. 3:**
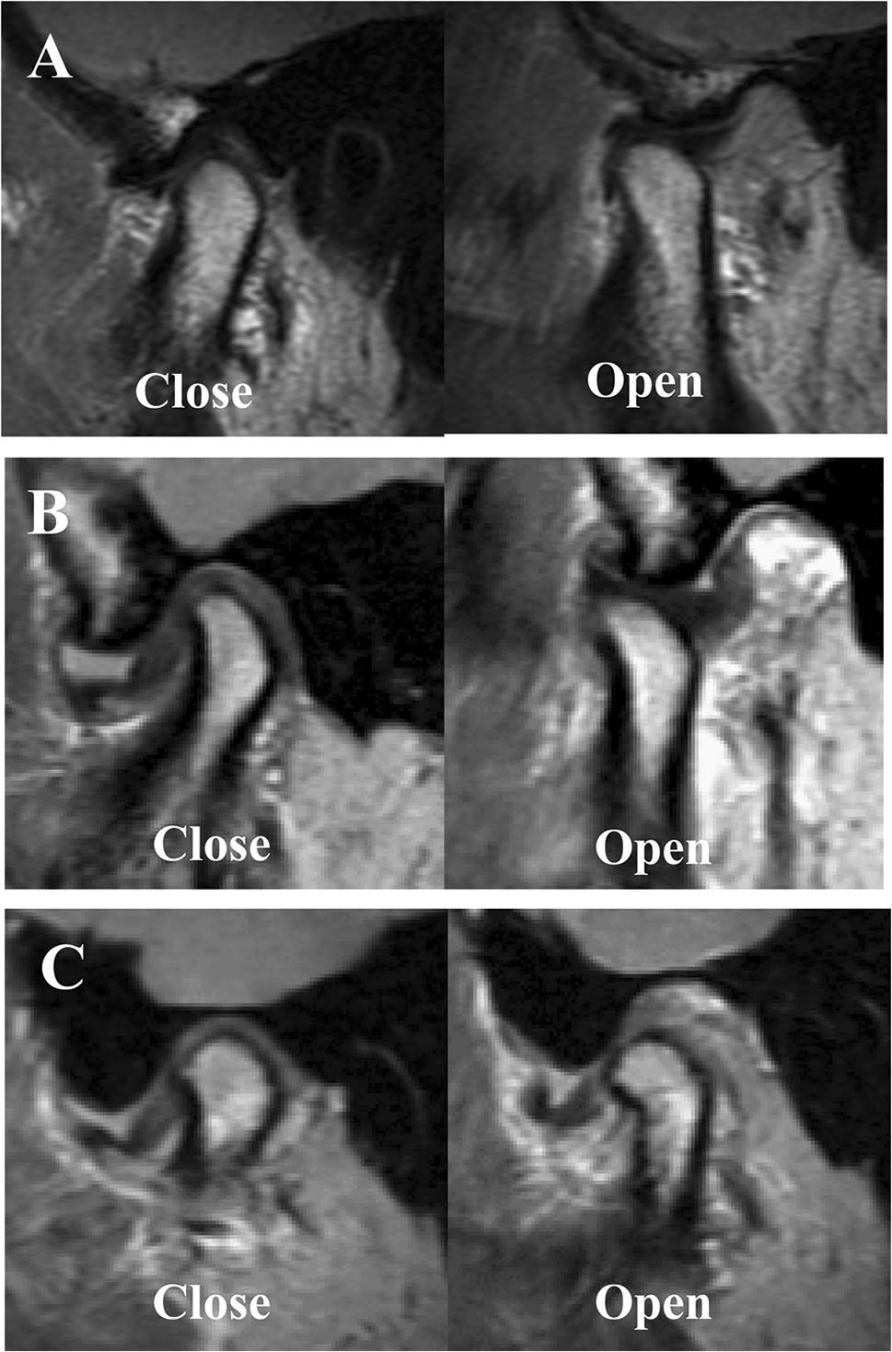
Dynamic disc abnormality in TMJ. **A** No dynamic disc abnormality. **B** Disc displacement with reduction. **C** Disc displacement without reduction

Dynamic articular disc abnormalities were classified into the following three groups [24]: no dynamic disc abnormality (Fig. 3A), in which articular disc displacement was not observed when the mouth was closed; articular disc displacement with reduction (DDwR, Fig. 3B), in which the articular disc was displaced when the mouth was closed, but the mandibular condyle captured the articular disc, and the relationship between the mandibular condyle and mandibular fossa appeared normal when the mouth was open; and articular disc displacement without reduction (DDwoR, Fig. 3C), in which the articular disc was displaced when the mouth was closed, and the posterior thickened part of the articular disc appeared to be located anterior to the anterior surface of the mandibular condyle in at least one sagittal plane when the mouth was open (alternatively, it had the appearance of residual sideways displacement when the mouth was open).

The Kappa coefficient was used to analyze the inter-rater reliability for the presence of dynamic disc displacement and OA. When a diagnosis differed between radiologists, a discussion for the definitive diagnosis was held, and a final decision was made by mutual agreement.

### Genotyping

DNA samples were collected non-invasively from the buccal mucosa of the subjects using FLOQSwabs (COPAN Diagnostics Inc.). Each of the patients was asked to obtain two buccal swabs from bilateral sides of the cheek. The examiner scraped the inside of cheek with the swabs firmly more than six times. Then, the swabs were stored in the freezer at − 20 °C. The obtained DNA samples were used to analyze the FTO gene (rs8044769). Genotyping was performed blindly by laboratory personnel (Takara Bio Inc.), and its accuracy was validated in advance using samples from another 22 cases, which showed a degree of agreement of 100%.

- (dependent variable: the presence of OA) and the predictors
- (covariates: age, sex, dynamic disc state [the presence of woR])

### Statistical analysis

The polymorphisms (rs8044769) were categorized into CC, CT, or TT. The age of the patients on the day of MRI was used in the statistical analysis. The descriptive statistics for the TMJs with OA and the TMJs without OA were evaluated using Fisher’s exact test and the Mann–Whitney *U* test. The BMIs of patients harboring different alleles of FTO polymorphisms were compared using the Kruskal–Wallis test. Furthermore, binomial logistic regression analysis was performed with the dependent variable being the presence or absence of OA, and the explanatory variables were sex, age, BMI, the rs8044769 genotypes (CC, CT, or TT), the presence of positional disc abnormalities, and the presence of disc displacement without reduction. Binomial logistic regression analysis was performed for each mandibular condyle to consider the effect of the laterality of the mandibular condyle. Model fitness was evaluated by using the Hosmer–Lemeshow test. The data were analyzed using the Statistical Package for Social Science (SPSS) version 23.0.

## Results

Inter-rater reliability for the presence of dynamic disc displacement and OA in 314 TMJs was excellent (*κ* = 0.92 and 0.82, respectively). Sixty-two osteophytes, 22 erosions, 38 subchondral cysts, and 47 atrophic deformities were diagnosed by MRI from the 324 TMJs. Consequently, the numbers of TMJs with OA and TMJs without OA were 113 and 211, respectively. The results of the descriptive statistics for the TMJs with OA and the TMJs without OA are shown in Table 1. The median age and BMI of the TMJs with OA were 59 years (interquartile range [IQR] 51–66) and 21.4 kg/m^2^ (IQR 18.8–23.7), respectively, whereas those of the TMJs without OA were 58 years (IQR 47–66) and 21.4 kg/m^2^ (IQR 19.1–23.7), respectively, showing no statistically significant difference between them. In addition, the TMJs with OA and the TMJs without OA showed no statistically significant differences in the distribution of sex. A Fisher’s exact test showed a statistically significant difference in the allele frequency of FTO gene polymorphisms between the TMJs with OA and the TMJs without OA (*P* = 0.011). In addition, there was a statistically significant difference in the distribution of positional disc abnormalities and dynamic disc abnormalities between the TMJs with OA and the TMJs without OA (*P* < 0.001).

**Table 1:** Descriptive statistics for TMJs with OA and TMJs without OA. *OA* osteoarthritis, *IQR* interquartile range, *AR* adjusted residual, *DDwR* disc displacement with reduction, *DDwoR* disc displacement without reduction

	TMJs with OA (*n* = 113)	TMJs without OA (*n* = 211)	*P*
Age [IQR]	59 [51–66] years	58 [47–66] years	0.426
Female *n* [%]	96 [85.0]	164 [77.7]	0.077
BMI [IQR]	21.4 [18.8–23.7] kg/m^2^	21.4 [19.1–23.7) kg/m^2^	0.702
rs8044769 *n* [AR]			
CC	74 [3.0]	102 [− 3.0]	0.011
CT	29 [− 2.6]	85 [2.6]	
TT	10 [− 0.7]	24 [0.7]	
Positional disc abnormality *n* [AR]			
Normal	1 [− 7.0]	74 [7.0]	< 0.001
Sideways	10 [− 3.1]	48 [3.1]	
Partial anterior	2 [− 2.1]	15 [2.1]	
Partial anterior and sideways	3 [− 3.4]	31 [3.4]	
Complete anterior	93 [11.5]	35 [− 11.5]	
Posterior	4 [− 0.1]	8 [0.1]	
Dynamic disc abnormality *n* [AR]			
Normal	1 [− 7.0]	74 [7.0]	< 0.001
DDwR	4 [− 6.8]	81 [6.8]	
DDwoR	108 [11.8]	56 [− 11.8]	

The median BMIs of patients in the CC, CT, and TT allele groups were 21.8 (IQR 19.1–24.1), 21.4 (IQR 19.1–22.8), and 19.6 (IQR 18.1–22.0) kg/m^2^, respectively, showing no significant differences among the groups (*P* = 0.116).

Furthermore, logistic regression analysis indicated that the CC allele of rs8044769 is a risk factor for OA, in contrast with the TT allele (Table 2; odds ratio = 2.765). Moreover, the presence of disc displacement without reduction was significantly associated with OA. The Hosmer–Lemeshow test showed the goodness of fit of the model adjusted for sex, age, BMI, and dynamic disc abnormality (χ^2^ = 6.266, df = 8, *P* = 0.617).

**Table 2:** The result of binary logistic analysis for associations between FTO polymorphism (rs8044769) and OA (*n* = 324TMJs). Hosmer–Lemeshow test: χ^2^ = 6.266, df = 8, *P* = 0.617. *OA* osteoarthritis, *BMI* body mass index, *PDA* positional disc abnormality, *DDwR* disc displacement with reduction, *DDwoR* disc displacement without reduction, *OR* odds ratio

	*B*	S.E	Sig	OR	95% CI for OR
Age	0.020	0.013	0.103	1.021	0.996–1.046
Sex	0.053	0.462	0.908	1.055	0.427–2.608
BMI	0.029	0.052	0.581	1.029	0.930–1.139
Normal vs DDwR	1.334	1.134	0.240	3.795	0.411–35.057
Normal vs DDwoR	5.121	1.032	< 0.001	167.544	22.172–1266.075
CC vs TT (rs8044769)	1.017	0.495	0.040	2.765	1.049–7.288
CT vs TT (rs8044769)	0.474	0.525	0.367	1.606	0.574–4.499

## Discussion

The involvement of gene polymorphisms in the development of OA in the knee and hip joints has been reported, and the discovery of risk-associated genes has attracted broad attention as they expand the possibilities of new treatments for OA [8, 25]. One such gene is the FTO gene, which has been reported to be associated with obesity [2, 26]. Although the structure of FTO has been elucidated, its exact function remains unknown [27]. A genome wide association study reported that rs8044769 in the FTO gene is associated with OA in the knee and hip joints [12]. However, Panoutsopoulou et al., who adjusted for the effects of BMI in a meta-analysis, reported that variations in FTO SNPs (rs8044769) were not associated with the presence of OA in the knee and hip joints [13]. In addition, Dai et al. provided evidence that rs8044769 in the FTO gene is not associated with either the risk of OA or BMI in the Chinese population [28]. However, traditional studies cannot determine whether obesity causes OA or whether weight gain is caused by reduced activity due to OA. Therefore, in the present study, we investigated the effects of the FTO gene polymorphism rs8044769 on OA in the TMJ, which is minimally affected by BMI. Logistic regression analysis showed no significant association between BMI and the presence of OA, and it suggested that the rs8044769 variant is significantly associated with TMJOA. However, results of an in vivo mouse model suggested that the development of pathological changes in TMJ could be caused by the excessive compressive mechanical force and high-fat diet-induced obesity [29]. In addition, it has been reported that a quick eating rate is positively associated with excess body weight [30]. Furthermore, several chewing features such as chewing pace and time are associated with obesity in young adolescents, and they might affect the development of the TMJ [31]. Thus, further investigation will be required to reveal the relationship among jaw mastication, obesity, and TMJOA.

Internal derangement has been categorized as normal → DDwR → DDwoR → OA. However, we support the recent protocol by Schiffman et al. which assesses the disc displacement and degenerative bone change in the condyle independently [32]. Therefore, in the present study, each TMJ was diagnosed, e.g., DDwR with OA or DDwoR without OA. Not a few patients have asymmetrical TMJs, for example, DDwoR with OA on the right side and DDwR without OA on the left side, and if the statistical analysis is calculated in units of patients, it is difficult to evaluate the association between disc abnormalities and OA. Hence, the present logistic regression analysis was performed in units of TMJs. Consequently, in this study, an association between rs8044769 and OA was observed even after adjusting for the effects of dynamic articular disc abnormalities, which have been reported to be strongly associated with TMJOA [33, 34]. Thus, the FTO gene might enhance bone metabolism dysfunction and promote the development of OA, triggered by disc displacement with reduction. These results indicate that the FTO gene could be involved in bone metabolism, as shown by abnormal finger development due to chromosomal duplication in the FTO locus region [35]. However, the present logistic regression analysis performed in units of TMJs may result in overestimation in the incidence rate for OA, and the action of rs8044769 might be more likely to occur in the TMJ than in other joints. In addition, the position of the disc was evaluated in a subjective way by two operators, but objective measurements to define the TMJ disc position were not performed. Yet, the operators had excellent agreement.

Early-onset OA is associated with a strong familial representation of disease. In contrast, the common late onset OA is caused by additive effects of genetics and environmental factors [28]. Since the FTO gene is likely to be involved in late onset OA, subjects under 30 years of age were excluded to minimize the possibility of early onset OA. However, the time of onset of OA cannot be determined from the results of this cross-sectional study, and patients with a history of early onset OA might have been included in the present study. Moreover, the prevalence and severity of OA vary according to race and ethnicity, and geographical and environmental factors also have a significant impact. Thus, further research is required to investigate the potential mechanisms underlying OA. The major barrier to genetic studies of OA is the need to obtain a large number of individuals diagnosed with OA with standardized imaging examinations. The availability of TMJ MRI and CT scans poses a major obstacle, and few studies on TMJOA and gene polymorphisms have been reported [36]. In particular, data on normal TMJ in the elderly are invaluable as a control, but their extraction is challenging. Our research group possesses a large MR database of TMJ data, from which sufficient control individuals’ data could be extracted for this study. On the other hand, several patients with normal disc without OA were extracted from the MR data set, and eventually, only one TMJ has a normal disc with OA among all the subjects, and the odds ratio of the presence of DDwoR in the binominal logistic regression analysis was significantly higher than those of the previous reports [34, 37]. In the present study, the categorical data of the dynamic disc abnormalities were input as explanatory variables to adjust for their effects on OA, and thus, the odds ratio of the presence of DDwoR should not be referred to as a risk assessment parameter.

The present cross-sectional study supported the evidence that genetic risk factors might be associated with the development of TMJOA in the Japanese population without involvement from the effect of BMI. However, large-scale longitudinal trials in more diverse and generalized populations will be needed to reveal details of such genetic involvement. In the future, the identification of genetic risk factors that influence the therapeutic outcome in OA is expected to lead to preoperative diagnostic techniques for the selection and provision of an optimal treatment method suitable for the patient’s genetic predisposition. The findings of this study also suggest the mechanism underlying the development of TMJOA and its evolutionary significance. In the future, these are expected to contribute to the understanding of degenerative diseases of the skeletal system caused by genomic variation, their treatment, and genome-based drug discovery for skeletal regeneration.

## Conclusions

We concluded that rs8044769 in the FTO gene could be involved in the development of TMJOA. Within the limitations of this retrospective study, the present study will allow for a deeper understanding of the pathophysiology of the TMJOA and supports the use of genetic risk factors for the preoperative examination and evaluation of therapeutic outcomes in this condition.

## Supplementary Information

Below is the link to the electronic supplementary material.

Supplementary file1 (PDF 330 KB)

## References

[1] Gerken T, Girard CA, Tung YC, Webby CJ, Saudek V, Hewitson KS, Yeo GS, McDonough MA, Cunliffe S, McNeill LA, Galvanovskis J, Rorsman P, Robins P, Prieur X, Coll AP, Ma M, Jovanovic Z, Farooqi IS, Sedgwick B, Barroso I, Lindahl T, Ponting CP, Ashcroft FM, O’Rahilly S, Schofield CJ (2007). The obesity-associated FTO gene encodes a 2-oxoglutarate-dependent nucleic acid demethylase. Science.

[2] Frayling TM, Timpson NJ, Weedon MN, Zeggini E, Freathy RM, Lindgren CM, Perry JR, Elliott KS, Lango H, Rayner NW, Shields B, Harries LW, Barrett JC, Ellard S, Groves CJ, Knight B, Patch AM, Ness AR, Ebrahim S, Lawlor DA, Ring SM, Ben-Shlomo Y, Jarvelin MR, Sovio U, Bennett AJ, Melzer D, Ferrucci L, Loos RJ, Barroso I, Wareham NJ, Karpe F, Owen KR, Cardon LR, Walker M, Hitman GA, Palmer CN, Doney AS, Morris AD, Smith GD, Hattersley AT, McCarthy MI (2007). A common variant in the FTO gene is associated with body mass index and predisposes to childhood and adult obesity. Science.

[3] Yang J, Loos RJ, Powell JE, Medland SE, Speliotes EK, Chasman DI, Rose LM, Thorleifsson G, Steinthorsdottir V, Mägi R, Waite L, Smith AV, Yerges-Armstrong LM, Monda KL, Hadley D, Mahajan A, Li G, Kapur K, Vitart V, Huffman JE, Wang SR, Palmer C, Esko T, Fischer K, Zhao JH, Demirkan A, Isaacs A, Feitosa MF, Luan J, Heard-Costa NL, White C, Jackson AU, Preuss M, Ziegler A, Eriksson J, Kutalik Z, Frau F, Nolte IM, Van Vliet-Ostaptchouk JV, Hottenga JJ, Jacobs KB, Verweij N, Goel A, Medina-Gomez C, Estrada K, Bragg-Gresham JL, Sanna S, Sidore C, Tyrer J, Teumer A, Prokopenko I, Mangino M, Lindgren CM, Assimes TL, Shuldiner AR, Hui J, Beilby JP, McArdle WL, Hall P, Haritunians T, Zgaga L, Kolcic I, Polasek O, Zemunik T, Oostra BA, Junttila MJ, Grönberg H, Schreiber S, Peters A, Hicks AA, Stephens J, Foad NS, Laitinen J, Pouta A, Kaakinen M, Willemsen G, Vink JM, Wild SH, Navis G, Asselbergs FW, Homuth G, John U, Iribarren C, Harris T, Launer L, Gudnason V, O’Connell JR, Boerwinkle E, Cadby G, Palmer LJ, James AL, Musk AW, Ingelsson E, Psaty BM, Beckmann JS, Waeber G, Vollenweider P, Hayward C, Wright AF, Rudan I, Groop LC, Metspalu A, Khaw KT, van Duijn CM, Borecki IB, Province MA, Wareham NJ, Tardif JC, Huikuri HV, Cupples LA, Atwood LD, Fox CS, Boehnke M, Collins FS, Mohlke KL, Erdmann J, Schunkert H, Hengstenberg C, Stark K, Lorentzon M, Ohlsson C, Cusi D, Staessen JA, Van der Klauw MM, Pramstaller PP, Kathiresan S, Jolley JD, Ripatti S, Jarvelin MR, de Geus EJ, Boomsma DI, Penninx B, Wilson JF, Campbell H, Chanock SJ, van der Harst P, Hamsten A, Watkins H, Hofman A, Witteman JC, Zillikens MC, Uitterlinden AG, Rivadeneira F, Kiemeney LA, Vermeulen SH, Abecasis GR, Schlessinger D, Schipf S, Stumvoll M, Tönjes A, Spector TD, North KE, Lettre G, McCarthy MI, Berndt SI, Heath AC, Madden PA, Nyholt DR, Montgomery GW, Martin NG, McKnight B, Strachan DP, Hill WG, Snieder H, Ridker PM, Thorsteinsdottir U, Stefansson K, Frayling TM, Hirschhorn JN, Goddard ME, Visscher PM (2012). FTO genotype is associated with phenotypic variability of body mass index. Nature.

[4] Stratigopoulos G, Padilla SL, LeDuc CA, Watson E, Hattersley AT, McCarthy MI, Zeltser LM, Chung WK, Leibel RL (2008). Regulation of Fto/Ftm gene expression in mice and humans. Am J Physiol Regul Integr Comp Physiol.

[5] van der Hoeven F, Schimmang T, Volkmann A, Mattei MG, Kyewski B, Rüther U (1994). Programmed cell death is affected in the novel mouse mutant Fused toes (Ft). Development.

[6] Peters T, Ausmeier K, Dildrop R, Rüther U (2002). The mouse Fused toes (Ft) mutation is the result of a 1.6-Mb deletion including the entire Iroquois B gene cluster. Mamm Genome.

[7] Fredriksson R, Hägglund M, Olszewski PK, Stephansson O, Jacobsson JA, Olszewska AM, Levine AS, Lindblom J, Schiöth HB (2008). The obesity gene, FTO, is of ancient origin, up-regulated during food deprivation and expressed in neurons of feeding-related nuclei of the brain. Endocrinology.

[8] Warner SC, Valdes AM (2017). Genetic association studies in osteoarthritis: is it fairytale?. Curr Opin Rheumatol.

[9] Peffers MJ, Balaskas P, Smagul A (2018). Osteoarthritis year in review 2017: genetics and epigenetics. Osteoarthr Cartil.

[10] Jeffries MA (2019). Osteoarthritis year in review 2018: genetics and epigenetics. Osteoarthr Cartil.

[11] Valdes AM, Spector TD (2011). Genetic epidemiology of hip and knee osteoarthritis. Nat Rev Rheumatol.

[12] Zeggini E, Panoutsopoulou K, Southam L, Rayner NW, Day-Williams AG, Lopes MC, Boraska V, Esko T, Evangelou E, Hoffman A, Houwing-Duistermaat JJ, Ingvarsson T, Jonsdottir I, Jonnson H, Kerkhof HJ, Kloppenburg M, Bos SD, Mangino M, Metrustry S, Slagboom PE, Thorleifsson G, Raine EV, Ratnayake M, Ricketts M, Beazley C, Blackburn H, Bumpstead S, Elliott KS, Hunt SE, Potter SC, Shin SY, Yadav VK, Zhai G, Sherburn K, Dixon K, Arden E, Aslam N, Battley PK, Carluke I, Doherty S, Gordon A, Joseph J, Keen R, Koller NC, Mitchell S, O’Neill F, Paling E, Reed MR, Rivadeneira F, Swift D, Walker K, Watkins B, Wheeler M, Birrell F, Ioannidis JP, Meulenbelt I, Metspalu A, Rai A, Salter D, Stefansson K, Stykarsdottir U, Uitterlinden AG, van Meurs JB, Chapman K, Deloukas P, Ollier WE, Wallis GA, Arden N, Carr A, Doherty M, McCaskie A, Willkinson JM, Ralston SH, Valdes AM, Spector TD, Loughlin J (2012). Identification of new susceptibility loci for osteoarthritis (arcOGEN): a genome-wide association study. Lancet.

[13] Panoutsopoulou K, Metrustry S, Doherty SA, Laslett LL, Maciewicz RA, Hart DJ, Zhang W, Muir KR, Wheeler M, Cooper C, Spector TD, Cicuttini FM, Jones G, Arden NK, Doherty M, Zeggini E, Valdes AM (2014). The effect of FTO variation on increased osteoarthritis risk is mediated through body mass index: a Mendelian randomisation study. Ann Rheum Dis.

[14] Pang H, Luo F, Dai F, Wu XH, Xu JZ (2013). Genome-wide association study for osteoarthritis. Lancet.

[15] Winburn AP, Stock MK (2019). Reconsidering osteoarthritis as a skeletal indicator of age at death. Am J Phys Anthropol.

[16] Isabel CA, Moysés MR, van der Bilt A, Gameiro GH, Ribeiro JC, Pereira LJ (2015). The relationship between masticatory and swallowing behaviors and body weight. Physiol Behav.

[17] de Leeuw R, Boering G, Stegenga B, de Bont LG (1995). Radiographic signs of temporomandibular joint osteoarthrosis and internal derangement 30 years after nonsurgical treatment. Oral Surg Oral Med Oral Pathol Oral Radiol Endod.

[18] Wilkes CH (1989). Internal derangements of the temporomandibular joint. Pathological variations. Arch Otolaryngol Head Neck Surg.

[19] Ruscitto A, Morel MM, Shawber CJ, Reeve G, Lecholop MK, Bonthius D, Yao H, Embree MC (2020). Evidence of vasculature and chondrocyte to osteoblast transdifferentiation in craniofacial synovial joints: implications for osteoarthritis diagnosis and therapy. Faseb j.

[20] Tasaki MM, Westesson PL (1993). Temporomandibular joint: diagnostic accuracy with sagittal and coronal MR imaging. Radiology.

[21] Westesson PL, Larheim TA, Tanaka H (1998). Posterior disc displacement in the temporomandibular joint. J Oral Maxillofac Surg.

[22] Katzberg RW, Westesson PL, Tallents RH, Anderson R, Kurita K, Manzione JV, Totterman S (1988). Temporomandibular joint: MR assessment of rotational and sideways disk displacements. Radiology.

[23] Foucart JM, Carpentier P, Pajoni D, Marguelles-Bonnet R, Pharaboz C (1998). MR of 732 TMJs: anterior, rotational, partial and sideways disc displacements. Eur J Radiol.

[24] Tasaki MM, Westesson PL, Isberg AM, Ren YF, Tallents RH (1996). Classification and prevalence of temporomandibular joint disk displacement in patients and symptom-free volunteers. Am J Orthod Dentofacial Orthop.

[25] van Meurs JB (2017). Osteoarthritis year in review 2016: genetics, genomics and epigenetics. Osteoarthr Cartil.

[26] Dina C, Meyre D, Gallina S, Durand E, Körner A, Jacobson P, Carlsson LM, Kiess W, Vatin V, Lecoeur C, Delplanque J, Vaillant E, Pattou F, Ruiz J, Weill J, Levy-Marchal C, Horber F, Potoczna N, Hercberg S, Le Stunff C, Bougnères P, Kovacs P, Marre M, Balkau B, Cauchi S, Chèvre JC, Froguel P (2007). Variation in FTO contributes to childhood obesity and severe adult obesity. Nat Genet.

[27] Han Z, Niu T, Chang J, Lei X, Zhao M, Wang Q, Cheng W, Wang J, Feng Y, Chai J (2010). Crystal structure of the FTO protein reveals basis for its substrate specificity. Nature.

[28] Dai J, Ying P, Shi D, Hou H, Sun Y, Xu Z, Chen D, Zhang G, Ni M, Teng H, Wang Y, Jiang Q (2018). FTO variant is not associated with osteoarthritis in the Chinese Han population: replication study for a genome-wide association study identified risk loci. J Orthop Surg Res.

[29] Du J, Jiang Q, Mei L, Yang R, Wen J, Lin S, Li H (2020). Effect of high fat diet and excessive compressive mechanical force on pathologic changes of temporomandibular joint. Sci Rep.

[30] Ohkuma T, Hirakawa Y, Nakamura U, Kiyohara Y, Kitazono T, Ninomiya T (2015). Association between eating rate and obesity: a systematic review and meta-analysis. Int J Obes (Lond).

[31] Idris G, Smith C, Galland B, Taylor R, Robertson CJ, Bennani H, Farella M (2021). Relationship between chewing features and body mass index in young adolescents. Pediatr Obes.

[32] Schiffman EL, Ahmad M, Hollender L, Kartha K, Ohrbach R, Truelove EL, Zhang L, Hodges JS, Sommers E, Anderson GC, Gonzalez YM, Guo X, Look JO (2017). Longitudinal stability of common TMJ structural disorders. J Dent Res.

[33] Campos MI, Campos PS, Cangussu MC, Guimaraes RC, Line SR (2008). Analysis of magnetic resonance imaging characteristics and pain in temporomandibular joints with and without degenerative changes of the condyle. Int J Oral Maxillofac Surg.

[34] Dias IM, Cordeiro PC, Devito KL, Tavares ML, Leite IC, TeschRde S (2016). Evaluation of temporomandibular joint disc displacement as a risk factor for osteoarthrosis. Int J Oral Maxillofac Surg.

[35] Stratakis CA, Lafferty A, Taymans SE, Gafni RI, Meck JM, Blancato J (2000). Anisomastia associated with interstitial duplication of chromosome 16, mental retardation, obesity, dysmorphic facies, and digital anomalies: molecular mapping of a new syndrome by fluorescent in situ hybridization and microsatellites to 16q13 (D16S419-D16S503). J Clin Endocrinol Metab.

[36] Liu J, Wang G, Peng Z (2020). Association between the MMP-1-1607 1G/2G polymorphism and osteoarthritis risk: a systematic review and meta-analysis. Biomed Res Int.

[37] Roh HS, Kim W, Kim YK, Lee JY (2012). Relationships between disk displacement, joint effusion, and degenerative changes of the TMJ in TMD patients based on MRI findings. J Craniomaxillofac Surg.

